# Relationships between restrained eating and personality and self-esteem

**DOI:** 10.1371/journal.pone.0313406

**Published:** 2024-11-26

**Authors:** John B. Nezlek, Catherine A. Forestell

**Affiliations:** 1 Center for Climate Action and Social Transformations (4CAST), Institute of Psychology, SWPS University, Warsaw, Poland; 2 Department of Psychological Sciences, College of William & Mary, Williamsburg, Virginia, United States of America; University Putra Malaysia: Universiti Putra Malaysia, MALAYSIA

## Abstract

We examined relationships between restrained eating and personality and self-esteem in a sample of 4382 undergraduates attending a university in the US, *M*_age_ = 18.9, *SD* = 1.42. Restrained eating was measured using the Dutch Eating Behavior Questionnaire, personality was measured using the BFI-44, and self-esteem was measured using the Rosenberg self-esteem scale. Restrained eating scores were regressed onto the five personality traits of the BFI-44 using ordinary least squares multiple linear regression. These analyses found that restrained eating was significantly and positively related to extraversion, conscientiousness, and neuroticism. Restrained eating was not significantly related to agreeableness and openness. Moderation analyses suggested that restrained eating was not related to neuroticism for women with obesity, but it was related for women without obesity. Restrained eating was negatively correlated with self-esteem, and this relationship was stronger for women than it was for men. These findings contribute to our understanding about the personality factors associated with restrained eating, which may help us better understand individual differences in dietary behaviors.

## Introduction

According to the Centers for Disease Control, between 2013 to 2016, about half of the American population attempted to lose weight, with around two-thirds of them attempting to do so by reducing their food intake [1]. Surveys of Europeans have found similar trends [e.g., 2]. In eating behavior research, the act of restricting one’s food intake is referred to as “restrained eating,” which is defined as the cognitive effort exerted to eat less than one desires [3].

Although restrained eating has often been conceptualized as problematic, even pathological, e.g., as a precursor or component of various eating disorders [4], ample research indicates that restrained eating is not necessarily pathological. As summarized by Schaumberg et al. [3] “Altogether, it appears that dietary restraint can prove a beneficial strategy for those attempting to control their weight, as it does not relate to increased levels of eating pathology when practiced as part of a well-validated weight management programme.” Such a conclusion is consistent with the fact that large percentages of people consciously restrain their eating over the long term but do not develop an eating disorder [5].

The present study examined relationships between restrained eating, as measured by the Dutch Eating Behavior Questionnaire [6], and personality, defined in terms of the Five Factor Model of personality [FFM; 7], and between restrained eating and self-esteem [8]. We examined relationships between restrained eating and the FFM because the FFM is frequently discussed as constituting the “building blocks” of personality, i.e., it is a comprehensive model of personality. We examined relationships between restrained eating and self-esteem because self-esteem is among the most widely studied individual difference variables in psychology.

We examined these relationships in a sample of 4,382 undergraduate students, 98% of whom were emerging adults (aged 18–29), with enough men (1,754) to provide a basis to compare men and women with adequate statistical power. Sampling a large number of men is important because men are under-represented in research on restrained eating. For example, in a recent review by Mills et al. [9] that examined relationships between personality and restrained eating, the majority of studies focused exclusively on women.

### Existing research: Personality

Under the assumption that individual differences in restrained eating represent non-pathological variability, the present study examined relationships between restrained eating and personality. When discussing previous research, we refer to measuring personality comprehensively, e.g., all five factors of the FFM vs. measures of a single factor or measures of constructs that concern a very specific individual difference such as perfectionism.

Although the results of existing research on relationships between restrained eating and personality are somewhat consistent, there are not many studies, and there are some shortcomings in this research. This is reflected in a recent review by Mills et al. [9] that included studies assessing restrained eating using four widely used measures: the DEBQ, the Three Factor Eating Questionnaire [TFEQ; 10], the Restraint Scale [RS; 11], and the Eating Disorder Examination Restraint subscale [EDE-Q-R; 12].

In terms of the DEBQ, of the seven studies cited by Mills et al., only four measured personality comprehensively, and none of these studied a non-clinical sample of both men and women. The state of the art was similar for research using other measures of restrained eating. Mills et al. reported no studies using the Restraint Scale that examined relationships between restrained eating and a comprehensive model of personality. For research using the TFEQ, only one study, Provencher et al. [13] used a comprehensive model of personality, and the sample was limited to women with obesity who were in an intervention study. Finally, only one study using the EDE-Q-R, Giovazolias et al. [14], used a comprehensive model of personality, and their sample consisted solely of women.

Putting these shortcomings aside, Mills et al. reported that neuroticism was positively related to restrained eating in one study using the RS and in one study using the TFEQ. Conscientiousness was positively related to restrained eating in two studies using the TFEQ, and one study using the DEBQ. Openness was positively related to restrained eating in one study using the DEBQ. Finally, agreeableness was not significantly related to restrained eating in any study using any measure.

Two studies that used the TFEQ that were not included by Mills et al. in their review found results that were partially similar to the results of the studies included by Mills et al. In a study of collegians, Sawhney, et al. [15], found that restrained eating as measured by the TFEQ was positively related to Conscientiousness, but not to any other of the FFM. In a study of morbidly obese patients accepted for bariatric surgery, Gade et al. [16] found that TFEQ cognitive restraint scores were negatively related to Neuroticism and were positively related to Agreeableness and Conscientiousness.

### Existing research: Self-esteem

Studies of relationships between restrained eating and self-esteem have used various measures of both constructs [9]. Although not all studies have found significant relationships between measures of these two constructs, with one exception [17], studies that have found significant relationships have found negative relationships between restrained eating and self-esteem. Regardless, the majority of studies of this relationship have examined samples of only women [9].

### Hypotheses and expectations

We expected that restrained eating would be positively related to conscientiousness and neuroticism. Conscientiousness is defined in terms of self-discipline, making and keeping plans, being organized, and so forth, all of which are required to restrain one’s eating consistently. Consistent with this, in their review of relationships between personality and food intake, Lunn et al. [18] concluded that “…the hypothesis that higher Conscientiousness may predict adoption of healthy dietary and other lifestyle recommendations appears to be supported.” Neuroticism includes anxiety and worry, and given that restraining one’s eating reflects concerns about one’s weight, restrained eating should be positively related to neuroticism.

Broadly speaking, existing research supports these two expectations [9]. Although significant relationships were not found in all studies examining these relationships, only two studies found a significant relationship in the direction opposite to those just described. Elhag and Morey [19] and Gade et al. [16] found a negative relationship between neuroticism and restrained eating, although in both of these studies participants were patients who were being treated for obesity.

We expected that self-esteem would be negatively related to restrained eating. Conceptually, assuming that the motivation to restrain one’s eating represents some type of attempt to improve some aspect of the self (e.g., appearance), people who have less positive self-evaluations should be more concerned about improving themselves by restraining their eating (losing weight). Consistent with this, previous research has found primarily negative relationships between self-esteem and restrained eating [9].

We also examined how the above relationships varied as a function of participants’ sex and BMI/obesity status. Given the lack of relevant theory and existing, we examined such possibilities on an exploratory basis.

## Method

### Participants

Participants were 4,382 undergraduate students who took an introductory psychology course at a university in the USA. The average age of participants was 18.9 years (*SD* = 1.42), and 60% were women. The data were collected over eight semesters, and over this time, response options for describing sex changed. For the first three semesters there were two options, male and female. For the next two semesters, there were three options, male, female, and transgender. For the last two semesters, there were four options, male, female, transgender, and other. Regardless, for the five semesters for which more options than male or female were available, 13 participants (less than 1%) selected an option other than female or male when describing themselves. Approximately two-thirds (64.7%) of participants described themselves as White/Caucasian, 7.9% were Black/African American, 15.1% were Asian/Asian American, 7.0% were Hispanic, 2.6% were Middle Eastern, 1.4% were Native American, and 1.4% did not describe themselves as belonging to any of these racial/ethnic groups.

Data were collected between: 2 September and 20 November, 2013; between 27 January and 30 May, 2014; between 13 September and 14 December, 2014; between 24 February and 9 May, 2015; between 23 September and 11 December, 2015; between 19 February and 9 May, 2016; between 19 September and 12 December, 2016; and between 20 September and 9 December 2017.

### Ethical statement

The study was conducted in accordance with the Declaration of Helsinki regarding the rights of research participants. Individuals voluntarily participated in partial fulfillment of a course requirement. Participants provided informed consent and were informed that they could refuse to answer any question without penalty. Participants consented in writing (electronically) by clicking on a link indicating their agreement to participate after being told that their names would not be associated with their answers and that they could terminate participation at any time without penalty. Consistent with these instructions, responses were de-identified prior to analysis.

The research protocols were approved by the Protection of Human Subjects Committee, College of William & Mary. Some protocols covered two semesters. The protocols were: PHSC-2017-09-18-12357-ccconway; PHSC-2016-09-12-11427-jbnezl; PHSC-2016-02-12-10920-jbnezl; PHSC-2015-09-07-10585-jbnezl; PHSC-2015-02-17-10114-pmvish; PHSC-2014-09-04-9771-pmvish; and PHSC-2014-01-21-9252-cldickter. Copies of these approvals are available as supporting information files. Copies of verbatim informed consent are available for all semesters.

### Inclusivity in global research

Additional information regarding the ethical, cultural, and scientific considerations specific to inclusivity in global research is included as supporting information, S1 Checklist: Inclusivity in global research questionnaire.

### Measures

Participants completed the BFI-44 [20], a measure of the Five Factor Model (FFM) of personality. The BFI-44 measures extraversion, agreeableness, conscientiousness, openness, and neuroticism. Participants responded to each of the 44 questions on the BFI-44 using the standard five-point scale with endpoints labeled 1 = strongly disagree and 5 = strongly agree. Scale scores were defined as the mean responses to the items constituting each scale [20].

Participants also completed the restrained eating subscale of the Dutch Eating Behavior Questionnaire [6] which contains 10 items. Participants responded to each item using a five-point scale with endpoints labeled 1 = seldom and 5 = very often. Scores were defined as the mean response to these 10 items, and higher scores indicated a stronger tendency to exhibit restrained eating.

Participants also indicated their current weight and height. Based on self-reported height and weight, we calculated participants’ body mass index following standard procedures [BMI; 21]. We also calculated a variable representing whether participants had obesity or not, defined as having a BMI greater than 29.99.

#### Overview of analyses

Our primary analysis was an ordinary least squares multiple regression in which restrained eating scores were regressed onto the scores of the BFI-44. On an exploratory basis, we examined if any significant relationships found in this analysis were moderated by participant BMI/obesity status and participant sex. This was done with a series of analyses using the PROCESS macro [22]. PROCESS can examine the moderation of only one relationship at a time. Accordingly, in these analyses, restrained eating scores were the outcomes, individual predictors were scores of the factors that were significant in the regression analyses and the moderators were sex (dummy-coded, 0 = not woman, 1 = woman), BMI scores, and a dummy-code representing whether participants had obesity (BMI > 29.99). In these analyses, measures of personality that were not the predictor were entered as covariates. Statistical significance was set at *p* < .05 for the regression and PROCESS analyses.

### Statistical power

The present sample provided power of functionally 1.0 to detect relationships less than Cohen’s f^2^ = .01 (a small effect) for the regression analyses we conducted [23].

## Results

### Descriptive statistics and correlations

First, we present descriptive statistics for the measures and the correlations between them in Table 1. All scales had moderate (.61 to.80) or substantial (.81 and above) reliability according to guidelines suggested by Shrout [24]. At the zero-order, restrained eating was significantly correlated with extraversion, conscientiousness, neuroticism, agreeableness, and with self-esteem. Of the 4247 participants who provided height and weight data, 200 were classified as having obesity.

**Table 1:** Descriptive statistics for, and correlations between, study variables.

	*M*	*SD*	*α*	n	BMI	SE	E	A	C	N	O
Restrained Eating	2.42	.94	.93	4382	.205	-.202	.054	-.032	.045	.211	-.014
BMI	22.88	3.87	n/a	4247		.005	.049	-.040	-.030	-.009	-.026
Self-esteem (SE)	3.70	.71	.88	3992			.338	.239	.335	-.584	.071
Extraversion (E)	3.20	.90	.88	4382				.131	.104	-.240	.181
Agreeableness (A)	3.78	.63	.77	4382					.255	-.263	.121
Conscientiousness (C)	3.52	.69	.82	4382						-.221	.032
Neuroticism (N)	3.01	.80	.83	4382							-.046
Openness (O)	3.69	.61	.78	4382							

Note: For *n* = 4382, 4247: |*r*| >.030 significant at *p* < .05; |*r*| >.039 significant at *p* ≤.01. All correlations involving self-esteem were significant at *p* < .01.

### Restrained eating and personality

Next, we regressed restrained eating scores onto scores for the five factors and sex (coded men = 0; women = 1). The overall model was significant, *F*(6,4368) = 87.50, *p* < .001, *R*^*2*^ = .107. Similar to the significant zero-order correlations we found, restrained rating was significantly related to extraversion (β = .091, *t* = 6.09, *p* < .001), conscientiousness (β = .056, *t* = 3.69, *p* < .001), and neuroticism (β = .186, *t* = 11.47, *p* < .001). Restrained eating was marginally significantly related to agreeableness (β = -.029, *t* = 1.89, *p* = .059) and was not significantly related to openness (β = -.025, *t* = 1.39 *p* = .165). Finally, the effect for sex was significant (β = .218, *t* = 14.30 *p* < .001). The mean restrained eating score for women (2.63) was higher than it was for men (2.11).

Next, we examined whether the significant relationships from the regression analysis were moderated by participant BMI, obesity status, and sex. This was done using the PROCESS macro [22]. When sex, BMI, and obesity status were examined separately, we found no significant or near significant moderation of relationships between restrained eating and extraversion, conscientiousness, or neuroticism.

**Fig 1:**
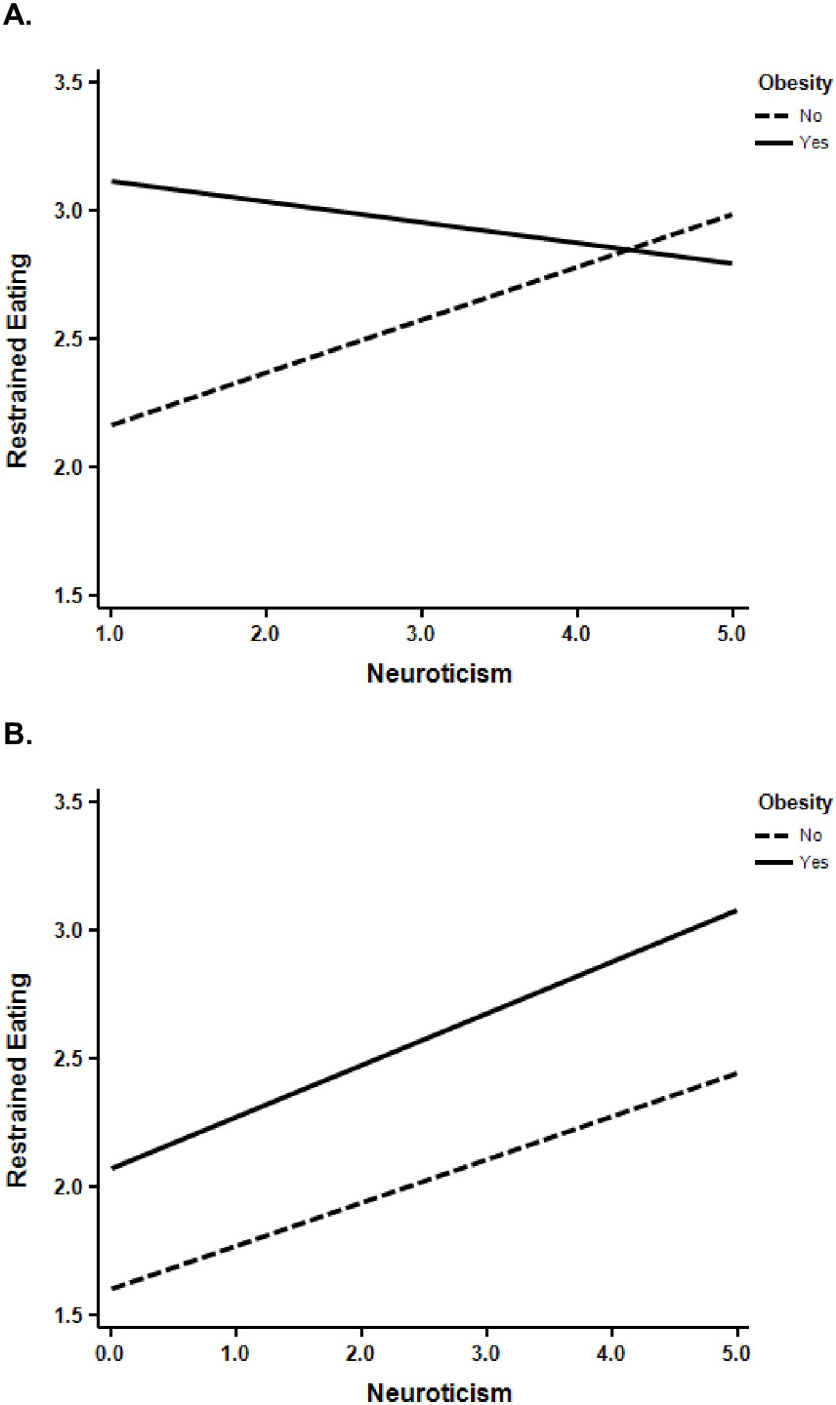
The moderating effect of obesity on the relationship between neuroticism and restrained eating. (A) Moderating effect for women. (B) Moderating effect for men. Solid lines represent those with obesity and dashed lines represent those without obesity.

When sex and obesity status were considered simultaneously (including their interaction), the analyses suggested that the relationship between restrained eating and neuroticism was moderated by the interaction of sex and obesity status, coefficient = -.306, *t* = 1.78, *p* = .076. Although this interaction did not reach standard levels of significance, we explored this interaction further by examining how relationships between restrained eating and neuroticism varied as a function of obesity status separately for men and women. The conditional effects from the PROCESS analysis indicated that restrained eating was positively related to neuroticism for men regardless of their obesity status: without obesity, coefficient = .207, *t* = 7.136, *p* < .001; with obesity, coefficient = .232, *t* = 1.95, *p* = .052. In contrast, for women, the relationship between restrained eating and neuroticism varied as a function of obesity status: for women without obesity the relationship was positive, coefficient = .238, *t* = 9.68, *p* < .001), whereas for women with obesity, the relationship was not significant, coefficient = -.043, *t* < 1. This interaction is depicted in Fig 1. There was no such interaction in the analyses of extraversion and conscientiousness.

It is noteworthy that if we had studied women only or if we had analyzed men and women separately, obesity status would have moderated the relationship between restrained eating and neuroticism (*p* = .025). The test we used examined if this moderation differed between men and women, which it did not, at least at the.05 level.

### Restrained eating and self-esteem

As expected, restrained eating and self-esteem were negatively correlated (Table 1). We also examined if this relationship was moderated by sex, BMI, obesity status, or a combination of these. These analyses found that sex moderated the relationship between self-esteem and restrained eating, coefficient = -.103, *t* = 2.53, *p* = .011. Although the negative relationship between self-esteem and restrained eating was significant for both men and women, it was stronger for women than it was for men. For women the coefficient was -.276 (*t* = 10.34, *p* < .001), and for men it was -.173 (*t* = 5.65, *p* < .001). The relationship between restrained eating and self-esteem was not moderated by BMI or obesity status or the interaction of these variables with participant sex.

## Discussion

The results of the analyses were consistent with our expectations. We found that restrained eating was positively related to conscientiousness and neuroticism. In terms of conscientiousness, restraining one’s eating requires self-discipline, which is an important component of conscientiousness. The motivation to restrain one’s eating also reflects concerns about one’s weight, and such concerns reflect aspects of neuroticism. Although these relationships are consistent with previous research, what makes the present results noteworthy is that we had a large nonclinical sample of both men and women. Many previous studies have examined such relationships among samples of only women, or small samples of men and women. Therefore, the present results meaningfully extend our understanding of restrained eating as it exists among men.

We also found that restrained eating was positively related to extraversion. One possible explanation for this relationship relies upon the facts that extraversion refers to the social domain (including assertiveness) and that eating is typically/often a social activity [25]. Therefore, restraining one’s eating may involve a social component. This could include refusing offers of food at social gatherings, declining invitations to join colleagues for meals, and so forth. Such refusals and declinations may require people to be assertive in the face of pressure from colleagues, who may be well-intended. Consistent with this, one item on the DEBQ explicitly concerns refusing food that is offered: “How often do you refuse food or drink offered because you are concerned about your weight?” This result is also consistent with results of Elhag and Morey [19] who found that restrained eating as measured by the DEBQ was positively related to extraversion.

Also consistent with our expectations, we found that self-esteem was negatively related to restrained eating, and this effect was stronger for women than for men. These findings are somewhat similar to those reported by Tiggeman [26] who found that self-esteem was negatively correlated with restrained eating for women, but restrained eating and self-esteem were not correlated for men. Tiggeman also reported that subjective feelings of being overweight were negatively related to self-esteem for women, but not for men. Taken together, these findings suggest that compared to women with higher self-esteem, those with lower self-esteem may be trying harder to restrain their eating to lose weight to improve their physical appearance in an attempt to reduce their body dissatisfaction. It is important to note that although the relationship between restrained eating and self-esteem was weaker for men than it was for women in our sample, the relationship was significant for men, suggesting that the mechanisms underlying this relationship may also exist for men, albeit with a smaller influence than for women.

### Importance of studying men and women together

For whatever reasons, sex differences in relationships between restrained eating and personality have not been studied extensively. Even when samples have both men and women, sex differences have not always been examined [e.g., 27]. The one study of which we are aware that measured personality comprehensively and that included men and women, Elhag and Morey [19], found no sex differences, although participants all had obesity, which limits the generalizability of their findings.

We mention this to highlight the contribution of the present study. It is the only study of which we are aware to examine sex differences in relationships between personality and restrained eating using a comprehensive measure of personality in a non-clinical sample. Although we did not find sex differences in these relationships, our sample provided a basis to examine such relationships.

### Neuroticism, sex, and obesity

Although the interaction of neuroticism, sex, and obesity status we found in the analysis of restrained eating was not significant at *p* < .05 (it was significant at *p* = .076), we believe the results are sufficiently interesting to merit some discussion. The moderation effect represented the fact that neuroticism was not related to restrained eating for women with obesity, whereas it was positively related to restrained eating for women without obesity and for men regardless of their obesity status. As discussed previously, a positive relationship between neuroticism and restrained eating is consistent with the fact that neuroticism contains a component of worrying.

In a meta-analysis of research on personality and body image, Allen and Walter [28] concluded that neuroticism was positively correlated to concerns about body image. Therefore, it is possible that for men and for women without obesity, those who are more neurotic are more concerned about their body image, which in turn leads them to restrain their food intake. In contrast, for women with obesity, concerns about body image may be sufficient motivation to restrain their eating irrespective of their level of neuroticism. Such an explanation is speculative and verifying it requires further research.

### Causality

Much of the research on relationships between restrained eating and personality tacitly assumes that restrained eating is a manifestation of personality [e.g., 29]. Such an assumption makes sense in that personality traits are conceptualized as relatively stable individual differences, whereas restrained eating is defined in behavioral terms. Nevertheless, to our knowledge, causal relationships between restrained eating and personality have not been examined.

It is possible that restraining one’s food consumption leads to changes in personality. For example, the discipline required to follow a controlled diet may have “spillover” effects to other life domains, which may result in increased conscientiousness. It is also possible that causal relationships are bi-directional, as Beckers et al. [30] found in a study of relationships between restrained eating and self-esteem. Regardless, examining such relationships will require longitudinal studies in which restrained eating and personality are measured on multiple occasions.

### Strengths, limitations, and future directions

The present study had a large sample and used well-established measures, two characteristics that support the validity of our findings. Similar to many studies of restrained eating, our sample consisted primarily of emerging adults, and we cannot be certain that the relationships we found generalize to people of different ages. There is also the issue of differences among cultures in the relationships we studied. Although Markey et al. [27] found only minor differences in relationships between restrained eating and self-esteem among the eight countries they studied, more research on such possibilities (i.e., differences in cultural norms related to eating behavior and body size and shape) is needed.

We also conceptualized personality within the context of FFM. Conceptualizing personality in terms of other models such as Eysenck’s might have provided different results. For example, Eysenck’s model does not measure conscientiousness. Importantly, we measured restrained eating using the DEBQ. The DEBQ is one of a few measures of restrained eating in widespread use, and different measures may not measure similar constructs [31]. For example, the DEBQ identifies dieters who restrict their food intake consistently, whereas the Restraint Scale (RS) and the Eating Disorder Examination-Questionnaire (EDE-Q) identify dieters who attempt to restrict their food intake but are prone to give in to temptation and eat more. Therefore, if restrained eating had been measured with the RS or the EDE-Q, we may have found stronger correlations with neuroticism and weaker correlations with conscientiousness than were found in the present study. More research that examines relationships between personality and restrained eaters defined in different ways is needed to address such questions.

## Conclusions

We believe the present study is the first to examine relationships between restrained eating and personality and self-esteem in a sample of men and women with and without obesity. We found that restrained eating was significantly and positively related to extraversion, conscientiousness, and neuroticism, whereas restrained eating was not significantly related to agreeableness and openness. Restrained eating was negatively correlated with self-esteem, and this relationship was stronger for women than it was for men.

By studying a large sample of men and women using a comprehensive model, the present results complement and meaningfully extend existing research, which has not done this. We found few sex effects or effects for obesity status. These null results, which, similar to any null results, must be viewed cautiously, suggest that personality manifests itself in similar ways in men and women, who have or do not have obesity.

## Supporting information

S1 Checklist Inclusivity in global research questionnaire. (PDF)
